# Improvement of Drought Tolerance in Rice (*Oryza sativa* L.): Genetics, Genomic Tools, and the WRKY Gene Family

**DOI:** 10.1155/2018/3158474

**Published:** 2018-08-07

**Authors:** Mahbod Sahebi, Mohamed M. Hanafi, M. Y. Rafii, T. M. M. Mahmud, Parisa Azizi, Mohamad Osman, Rambod Abiri, Sima Taheri, Nahid Kalhori, M. Shabanimofrad, Gous Miah, Narges Atabaki

**Affiliations:** ^1^Laboratory of Climate-Smart Food Crop Production, Institute of Tropical Agriculture and Food Security, Universiti Putra Malaysia, 43400 Serdang, Selangor, Malaysia; ^2^Laboratory of Plantation Science and Technology, Institute of Plantation Studies, Universiti Putra Malaysia, 43400 Serdang, Selangor, Malaysia; ^3^Department of Land Management, Faculty of Agriculture, Universiti Putra Malaysia, 43400 Serdang, Selangor, Malaysia; ^4^Department of Crop Science, Faculty of Agriculture, Universiti Putra Malaysia, 43400 Serdang, Selangor, Malaysia; ^5^Department of Biochemistry, Faculty of Biotechnology and Biomolecular Sciences, Universiti Putra Malaysia, 43400 Serdang, Selangor, Malaysia; ^6^Department of Biology, Faculty of Science, Universiti Putra Malaysia, 43400 Serdang, Selangor, Malaysia; ^7^Iran Azad University of Tehran Science & Reserach Branch, Hesarak, Tehran 1477893855, Iran

## Abstract

Drought tolerance is an important quantitative trait with multipart phenotypes that are often further complicated by plant phenology. Different types of environmental stresses, such as high irradiance, high temperatures, nutrient deficiencies, and toxicities, may challenge crops simultaneously; therefore, breeding for drought tolerance is very complicated. Interdisciplinary researchers have been attempting to dissect and comprehend the mechanisms of plant tolerance to drought stress using various methods; however, the limited success of molecular breeding and physiological approaches suggests that we rethink our strategies. Recent genetic techniques and genomics tools coupled with advances in breeding methodologies and precise phenotyping will likely reveal candidate genes and metabolic pathways underlying drought tolerance in crops. The WRKY transcription factors are involved in different biological processes in plant development. This zinc (Zn) finger protein family, particularly members that respond to and mediate stress responses, is exclusively found in plants. A total of 89* WRKY *genes in* japonica *and 97 WRKY genes in* O. nivara* (OnWRKY) have been identified and mapped onto individual chromosomes. To increase the drought tolerance of rice (*Oryza sativa *L.), research programs should address the problem using a multidisciplinary strategy, including the interaction of plant phenology and multiple stresses, and the combination of drought tolerance traits with different genetic and genomics approaches, such as microarrays, quantitative trait loci (QTLs),* WRKY *gene family members with roles in drought tolerance, and transgenic crops. This review discusses the newest advances in plant physiology for the exact phenotyping of plant responses to drought to update methods of analysing drought tolerance in rice. Finally, based on the physiological/morphological and molecular mechanisms found in resistant parent lines, a strategy is suggested to select a particular environment and adapt suitable germplasm to that environment.

## 1. Introduction

As sessile organisms, plants encounter a wide spectrum of adverse conditions in their environment. Water deficit is a widespread challenge to sustainable agriculture [1]. Recently, a considerable body of research has developed around the theme of plant feedback under drought conditions. Evidence suggests that various pathways, mechanisms, and structures are among the most important factors in plant responses to dehydration. Water stress includes both water deficit and low-osmotic stress: desiccation and dehydration or drying and drought. Depending on the response, drought and desiccation tolerance occur* via* various mechanisms in plants.

Although desiccation tolerance is rare, it occurs globally [2]. Desiccation tolerance is found in plants, microorganisms, animals, mosses, terrestrial microalgae, symbiotic fungi, cyanobacteria (photobionts), yeast, and bacteria [3]. Drought tolerance is defined as plant tolerance under the minimum level of moisture content in the cytoplasm when the water content constitutes ~23% or ~0.3 g of the fresh and dry tissue, respectively. Drought tolerance mechanisms, including morphological adaptations, physiological acclimation, and cellular adjustments, are controlled by genetic factors at different steps. Morphological adaptations include increased root thickness and length, waxy or/and thick leaf coverings, decreased leaf weight and size, smaller epithelial cells, delayed leaf senescence, and increased green leaf area. Physiological acclimation consists of the following: higher stomatal density and conductance; decreased transpiration rates; reduced and early asynchrony between female and male flowering and maturation; and better production, accumulation, assimilation, and seed and biomass yield partitioning. Finally, cellular adjustments for desiccation tolerance entail increased chlorophyll content, particle numbers, and harvest index and lower osmotic potential [4]. In drought and desiccation tolerance, the accumulation of different molecules, such as nonreducing disaccharides and oligosaccharides, solutes with equal osmotic potential and particular proteins and tends to fluctuate [5]. The biosynthesis and interaction of drought and desiccation tolerance-related molecules are vital processes in living organisms under stress [6]. To overcome drought stress, which leads to crop loss worldwide, access to efficient protocols for phenotyping and to exotic germplasm is necessary [7].

Rice is the main staple food for one-third of people worldwide [8], providing up to 80% of these individuals' daily calories [9]. However, rice is considered one of the most drought-susceptible plants due to its small root system, thin cuticular wax, and swift stomatal closure [10]. Reportedly, nearly 23 million hectares of rain-fed rice face drought stress [11]. Genetic variations in the rice genes responsible for drought tolerance have been revealed* via* the screening and characterization of rice germplasm at different molecular, genetic, and morphological levels while under drought stress [12]. Hence, it may be possible to neutralize drought stress in rice in the future by developing drought-tolerant varieties.

Early senescence as a consequence of drought stress leads to an array of changes in many rice traits; tillering, leaf expansion, and midday photosynthesis are suppressed by drought stress because of early senescence [10]. Accumulation of osmolytes and organic acids, a reduction in photosynthetic efficiency, and changes in carbohydrate metabolism are the typical biochemical and physiological drought tolerance responses in rice [13]. In rice, reactive oxygen species (ROS) formation in various cellular compartments, such as mitochondria, peroxisomes, and chloroplasts, is an inevitable result of water shortage [14]. Overall, drought tolerance is a multifunctional output of numerous molecular, morphological, and biochemical characters. Although the mechanisms by which rice adapts to drought stress have been studied extensively, collectively, these data can also help understand the mechanism of the drought response and improve drought tolerance in rice [15]. Due to the availability of genome-wide molecular markers, genome sequence information, and inexpensive genotyping platforms in rice, it is currently possible to routinely apply marker-assisted breeding techniques to improve rice grain yield under drought stress. Moreover, different functional databases and genomic resources for rice, as well as the latest omics advances, have facilitated the characterization of genes and pathways involved in drought tolerance that help in candidate gene identification and allele mining [12]. To this end, the current review focuses on reported work on mechanisms and some effects of drought stress on rice yield, as well as management strategies to overcome the effects of drought on rice.

## 2. Quantitative Trait Loci for Drought Tolerance in Rice

The drought tolerance mechanism is complex, influenced by variations in plant phenology and controlled by several quantitative trait loci (QTLs) [16]. The plant response is complex and difficult to understand unless a thorough study of the genetic and physiological bases of these responses is conducted. Neither modern genetics nor traditional breeding can effectively improve the drought tolerance of crop plants if the molecular mechanisms correlated with seed yield stability are not well understood [17]. Advances in systematic plant genomics, phenotyping, and plant physiology lead to new discoveries in crop drought tolerance. Hence, crop breeders will be able to increase crop yields using the latest gene network information and tools for plant improvement [18]. As improving plant physiology enhances our knowledge of the complex drought tolerance network and its relation with different traits, successful genomics and molecular biology approaches and increased selection efficiency will result in the identification of candidate QTLs and genes related to these traits. Molecular breeding approaches can be used to exploit QTLs for crop improvement; thus, candidate genes are the main targets for genetic engineering and the production of transgenic lines [19].

The identification of the candidate genes responsible for plant tolerance under different abiotic stresses, along with the use of the most suitable promoters associated with these events, is essential to develop transgenic crops with enhanced drought stress tolerance [19]. Although genetic engineering approaches involve costly regulatory procedures and negative public perceptions of biosafety, “molecular breeding” and “Induced Local Lesions in Genome” (TILLING) products are widely accepted [19]. The QTLs are ordinarily part of linkage mapping or linkage analysis-based QTL mapping [20]. Traditional mapping of QTLs involves mapping populations in which traits correlated with drought tolerance are segregating; identifying polymorphic markers; genotyping the mapping populations* via* polymorphic markers; constructing genetic maps; accurately phenotyping based on drought tolerance-correlated traits; and finally, QTL mapping according to genotypic as well as phenotypic data. Several linkage mapping studies have been conducted on drought tolerance in different crops [16].

Because of inherent limitations associated with mapping populations, linkage analysis-based QTL mapping cannot offer detailed information about QTLs. These limitations include the following: the identified QTLs are commonly associated with large chromosomal segments or genomic regions due to insufficient time for recombination, inadequate phenotypic variation related to existing traits in the mapping population, and the segregation of different QTLs linked to the same traits in diverse mapping populations [89].

Linkage disequilibrium- (LD-) based association mapping (AM), which has been used in human genetics, has been suggested as a different QTL mapping approach to overcome some of these constraints in various crop species [90]. The association mapping (AM) method includes five steps: selecting various individual groups or panels from a natural germplasm/population pool; recording accurate phenotypic data on each group/panel; high-density sequencing of interesting candidate genes or the panel's genotyping markers; studying the level of genetic differentiation among panels within the particular population (population structure) and the relatedness coefficient between individual pairs within the population (kinship); and analysing the association mapping results according to data obtained on population structure, kinship, and the correlation of genotypic/haplotypic and phenotypic data. There are many advantages of the association mapping approach over biparental linkage mapping, as follows: higher resolution because of the utilization of all recombination events throughout the evolutionary history of a specific crop species; bypassing the development of a particular mapping population and the provision of a natural germplasm collection for a specific crop to reduce the required time for QTL mapping; using the same genotyping data and AM panel for mapping different traits mapping, making it a cost-effective approach; eliminating randomly recombinant inbred lines that express an insufficient agronomic type from the population's structure; and being able to sample and present many alleles per locus relative to linkage mapping (a survey of only two alleles). In summary, many QTLs related to drought tolerance in rice have been identified (Table 1). However, to date, only a few QTL studies on the impact of drought on grain quality have been reported; for example, a major QTL with an additive effect on grain yield under drought stress at the reproductive stage,* qDTY1.1*, was reported on rice chromosome 1 and flanked by RM11943 and RM431 in three populations: N22/Swarna, N22/IR64, and N22/MTU1010 [91]. Most QTLs have been identified based on a wide range of important traits, including (i) yield components, (ii) physiological responses, such as relative water content, osmotic potential, osmotic adjustment, leaf osmotic potential, flag leaf rolling index, chlorophyll contents, carbon isotope ratio, grain carbon isotope distinction, water-soluble carbohydrates, and (iii) root traits.

Several important mapped QTLs, as well as major genes related to abiotic stress tolerance, such as drought tolerance in rice and other crops, are available at (http://www.plantstress.com/biotech/index.asp?Flag=1). The QTLs identified for drought traits can only represent a small portion of phenotypic variation in crops. Thus, their direct use in marker-assisted selection (MAS) in breeding programmes is not sufficiently effective.

### 2.1. Analysis of QTLs Using Mathematical Models

Mathematical models in biology facilitate the understanding of yield efficiency as a multipart trait during drought. The procedure involves separating the phenotypes of the plants and their responses to environmental factors into fundamental and simple responses [92]. Modelling has been successfully used for leaf growth in maize [93]. Ecophysiological models and the precise measurement of environmental variables may address the problems posed by nonstable QTLs under different environmental conditions.

Combining ecophysiological models with QTL analysis predicts phenotypes that result from allelic combinations in each model. In this method, the QTLs related to a well-defined function and theories about the function of the gene underlying the QTL are expected to be more precise, and the number of candidate genes is lower than in conventional QTL mapping. Moreover, by using this approach, the reaction of a plant to a given environment can be predicted based on regulated gene networks [94]. Different pathway and network models can be developed using omics datasets that are activated in certain genotypes in response to drought. Models of the relationships between particular pathways and routes of the physiological responses to drought can be built directly using network information. The QTL cloning based on identified candidate genes will occur by comparing QTLs and gene regulatory networks for each parameter underlying the models. If all possible molecular and physiological aspects of a plant with the same germplasm in a particular drought situation are studied well, the phenotypes can be separated from the genes and the mechanisms, and suitable models can be built based on different allelic combinations.

### 2.2. Towards the Positional Cloning of QTLs Responsible for Drought Tolerance 

In general, QTLs identified by linkage mapping-based methods are located in 10 to 20 cM intervals, which have low resolution. Additionally, the interval of the QTL may span several candidate genes with causal influences on the trait. The positional cloning of QTLs has been undertaken in many crop species to identify casual genes [95]. Four main steps are involved in the cloning of QTLs: (i) restricting the region of the QTL using a mapping population, (ii) identifying a contig that spans the QTL region by screening for closely associated molecular markers in a very large insert library (e.g., a bacterial artificial chromosome library), (iii) sequencing the contig and identifying candidate genes, and (iv) confirming the candidate gene's effects on the phenotype. There are many reports on the cloning of QTLs related to different traits [96], but few address the cloning of QTLs linked to drought tolerance. For example, Vgt1, a flowering time QTL related to drought stress, was cloned in maize [96]; it showed an advantage in lowlands with warmer conditions by avoiding flowering at a phase when the plant would not have enough resources to reproduce successfully. Furthermore,* Vgt1 *affected the differentiation of maize varieties at different heights. A full subfamily G (HvABCG31) transporter gene encoding an ATP-binding cassette and affecting water protection in leaves was recently cloned from barley and rice [97]. The fine mapping of a drought response can isolate a QTL region spanning many genes. Genes with an expression pattern associated with drought stress or encoding enzymes or proteins involved in drought stress-responsive metabolic pathways would be strong candidates for additional experiments. The largest changes in gene expression and protein or metabolite profiles may be directly identified from the mapping population. Synchronization between loci that control gene expression (eQTL), proteins (pQTL), and metabolites (mQTL) and yield and physiologically related loci allows inference of the biochemical progression underlying the physiological responses. In addition, colocalization of physiological QTLs and eQTLs for different traits can help identify candidate genes responsible for drought tolerance and facilitate positional cloning [98]. Integrating mapping populations and gene expression profiles of the lines using Affymetrix microarrays permits the identification of* trans*-eQTLs and delivers valuable data on the regulatory networks underlying different drought tolerance mechanisms. Classifying populations based on microarray analysis is an expensive method, but comparing the transpiration efficiency (high or low) of the progeny lines reduces the cost [99]. Modern genomics methods, such as next-generation sequencing (NGS) and mapping, promise to accelerate the cloning of QTLs associated with drought tolerance traits. Consequently, cloning QTLs related to drought tolerance offers an opportunity to confirm candidate genes. Finally, these genes may be used to develop transgenic staple crops as well as other crop species.

Regardless, marker-assisted breeding is not a perfect tool because it is effective only with major QTLs; hence, minor QTLs would be underrepresented in the selection process, resulting in missed genetic gains. Therefore, genomic selection (GS) has been suggested as a technique to show the collective impact of all alleles on polygenic traits. The GS method is advantageous because it includes the minor genes from traditional MAS [100]. This method is a form of MAS that reveals the genetic variances in each individual according to estimated breeding values from a genomic dataset. Compared to phenotypic selection, GS reduces the selection time for most traits by half per cycle in* Arabidopsis*, barley, and maize [101]. The GS is also a perfect method for use with altered environmental conditions and rare molecular markers [102]. The estimated precision of the breeding values of maize using GS has been reported to be 0.58 for grain yield and is predicted to be a better choice than other techniques considering the annual genetic gain of maize [101, 103].

## 3. Identification of Candidate Drought Tolerance Genes in Model Plant Systems

A wide range of candidate genes responsible for drought tolerance can be identified* via *important advances in model plant species. To date, the genomes of many model plants, as well as major plant species, have been sequenced [104]. Genome annotation, functional genomics, and molecular physiology studies have been conducted in several model and major crops to identify candidate genes involved in drought tolerance. These candidate genes include a large family of genes expressed under drought stress. Different proteins expressed by drought stress-associated candidate genes play significant roles in (i) cellular protection, such as osmotic adjustment, structural adaptation, repair, degradation, and detoxification and (ii) positive interactions with other proteins and transcription factors, such as protein kinases and bZIP, MYB, and DREB, which are involved in plant drought responses by regulating other responsive genes, such as those involved in cell protection, to cope with drought stress in plants. Identifying drought tolerance (DT) genes from different model plants and major crops is vital to understanding the functional basis of the DT mechanism and its downstream use, including validation* via* MAS through molecular breeding. The transcriptomic responses of some candidate DT genes identified from different plant species have been characterized and evaluated (Table 2).

Candidate genes should be validated* via* approaches, such as expression analysis, qRT-PCR, incorporation into QTL maps, linkage mapping, TILLING and allele mining, and applications of these approaches have been reviewed previously [105]. In recent years, many transcriptomic and functional genomic studies have been conducted to understand the stress mechanisms in different crop plants. One common approach that effectively isolates the candidate genes responsible for drought stress in drought-resistant genotypes is the generation of expressed sequence tags (ESTs) from cDNA libraries (normalized or nonnormalized) of tissues collected under drought. To date, many drought-responsive genes have been identified from several crop species (Table 2). Normalized cDNA libraries from rice seedlings led to the identification of many genes responsible for drought tolerance that were highly expressed under drought [106].

Transcriptional profiling is another approach to identify candidate genes and includes the differential gene expression analysis of plant tissues at different times after the onset of drought stress, as well as between drought-susceptible and drought-tolerant genotypes [107]. However, selection of the correct tissue type, tissue stage, and stress treatment (i.e., timing and intensity) are essential to determine the minimum drought conditions for isolating RNA for transcriptomics studies [108]. Near-isogenic lines (NILs) are the gold standard for genetic materials; they vary only in the specific trait among genotypes with diverse genetic backgrounds. Hence, a focus on NILs can provide high-confidence results that are very specific and are obtained from differentially expressed genes related to the target traits. Additionally, miRNAs have been demonstrated to be involved in drought responses and tolerance in many crop plants, such as soybean and rice, and can improve drought tolerance in plants [109].

There are various platforms that can be applied for transcriptional profiling, including PCR-based differential display PCR (DDRT-PCR) [110], cDNA-amplified fragment length polymorphism (cDNA-AFLP) [111], cDNA and suppression subtractive hybridization (SSH) [112], cDNA and oligonucleotide microarrays [113], and digital expression analysis based on EST counts [114]. Super-serial analysis of gene expression (super-SAGE) is another technique that can be successfully used in different crops under stress conditions [115]. As NGS technology provides precise digital and real-time analysis of sequence-based transcriptomes, other methods, such as microarrays, will likely be replaced with NGS in the near future. The use of NGS in gene expression analysis has led to novel techniques such as DeepSAGE [116], Digital Gene Expression-TAG (DGE-TAG) [117], and RNA-Seq [118].

The application of RNA-Seq, which involves NGS technology, has several advantages in the examination of transcriptome structure, such as the discovery of splice junctions and allele-specific expression [119], and NGS may provide high-throughput sequencing results directly from the RNA of stress-challenged tissues from different genotypes. The transcriptional profiling of drought-tolerant and drought-sensitive genotypes can identify candidate genes responsible for drought tolerance and, in combination with genetic/QTL maps, can act as “genic molecular markers” [120].

Several candidate genes identified* via* the above processes may be related to QTLs for drought tolerance traits. Therefore, a genetics and genomics method that allows the quantitative analysis of transcriptional profiling may isolate the expression QTLs (eQTLs) for drought tolerance traits [105]. Accordingly, if expressed QTLs are detected in the* cis* condition, the genes of interest based on molecular markers will act as “diagnostic markers” for the particular traits [121]. The results of our recent NGS (transcriptome) study on the screening and identification of novel genes involved in the most drought-resistant Malaysian rice variety identified thousands of up- and downregulated novel genes involved in more than one hundred different pathways (personal communication, Sahebi et al. 2016). Transcriptional profiling based on NGS technology will likely be used to identify candidate drought tolerance genes from major crop species and subsequently utilize them in molecular breeding or genetics and genomics.

## 4. The Morphological Responses of Rice under Drought Stress

Under drought stress, plants experience high rates of transpiration and lack sufficient water near the roots. Drought significantly impairs the growth, development, and yield of rice. When water is lacking, rice typically stops or slows down its growth [64]. Rice growth and development decrease as a result of poor root growth; leaf surface traits are reduced, which impacts the radiation load on the leaf canopy, delays or reduces the rate of normal crop senescence as maturity is approached, and inhibits stem reserves [122]. Many studies have reported an array of early morphological changes in rice under drought [123]. Cell growth is impaired by decreases in turgor pressure under stress [124]. Drought impacts both expansion growth and elongation [125] and inhibits cell enlargement more than cell division [126]. Additionally, it inhibits rice seedling germination [127] and decreases the number of tillers [128, 129] and plant height [130]. A reduction in biomass production is a common adverse effect of drought stress [131]. Several studies have revealed a reduction in the dry and fresh weights of roots [10] and shoots [128] under drought. Reductions in fresh root and shoot weights and lengths ultimately reduce the biochemical processes and photosynthetic rate in rice [132].

### 4.1. The Effect of Drought Stress on Yield

**Figure 1:**
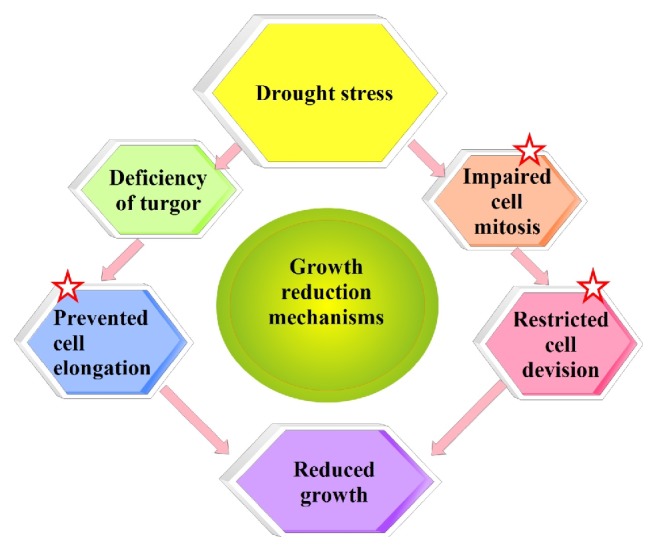
*Annotation* mechanisms of growth decline under drought stress condition. Under water deficit condition, cell elongation is hindered in higher plants by lessened turgor pressure. Decreased water uptake leads to reduce in tissue water contents. Consequently, turgor is missing. Similarly, water deficit condition also decreases the metabolites and photo assimilation required for the cell division. As a result, spoiled mitosis, cell elongation, and expansion lead to decreased growth.

The grain yield of rice severely decreases under drought stress [133]. Drought stress at the booting [134] and flowering stages disrupts floret initiation, leading to slow grain filling and spikelet sterility and resulting in reduced grain weight and poor paddy yield [135]. The most common characteristics of rice under drought stress include decreases grain weight and size [128], the 1000-grain weight, and seed-setting rate [10] and increases in spikelet sterility [136]. Water deficits restrict the grain filling period, resulting in in reduced grain yields [137]. Drought stress disrupts leaf gas exchange, limits the sizes of source and sink tissues, and impairs assimilate translocation and phloem loading [138]. Drops in yield may be due to reductions in CO2 assimilation rates induced by drought or decreases in photosynthetic pigments, stomatal conductance, stem extension, water use efficiency, the activities of starch and sucrose biosynthetic enzymes, and assimilate partitioning, resulting in reduced growth and productivity of the plant [139]. Drought duration and crop growth stage are two determinants of grain yield loss [140], as is the harshness of the drought stress [136]. Genetic, physiological, morphological, and ecological events and their multifaceted interactions are involved in cell division, enlargement, and differentiation and affect plant growth. Drought stress strongly affects the quantity and quality of plant growth through three main steps (Figure 1).

Cell growth is limited significantly due to reductions in turgor pressure under drought stress [124]. Cell elongation in higher plants under severe drought stress may be limited by disrupted water flow between the xylem and the surrounding elongating cells [141]. Reductions in mitosis, cell elongation, and cell expansion cause decreases in plant leaf area, height, and crop growth under water deficit [141, 142].

### 4.2. Global Strategies for Studying the Genetics of Drought Tolerance in Low-Yielding Areas

Molecular and physiological breeding approaches to improve drought tolerance in rice have not been very successful to date and suggest the need for a careful rethinking of our strategies. Some recently developed plant genomics platforms and methods may help overcome earlier limitations; the other techniques may have led us down incorrect paths. There are three main approaches to improve drought tolerance in rice and other crops: (i) experience-based selection for rice varieties with high yields under water deficit: this method has been widely used, and the excellent morphology of new cultivars is evidence of the success of this method. However, many studies have shown that the grain yield of these cultivars may not be sufficient to meet demand [143]; (ii) expression of physiological ideotypes for rice under drought to improve yield, then identifying sources of variation for these traits to introduce into selected varieties: in other words, genomics, breeding, and physiology approaches are integrated to improve drought tolerance in rice [144]. For this purpose, screening elite tolerance cultivars with high water use efficiency (WUE) using carbon isotopes is strongly suggested; and (iii) use of marker-assisted selection to screen for QTLs, including desirable alleles for drought tolerance.

**Figure 2:**
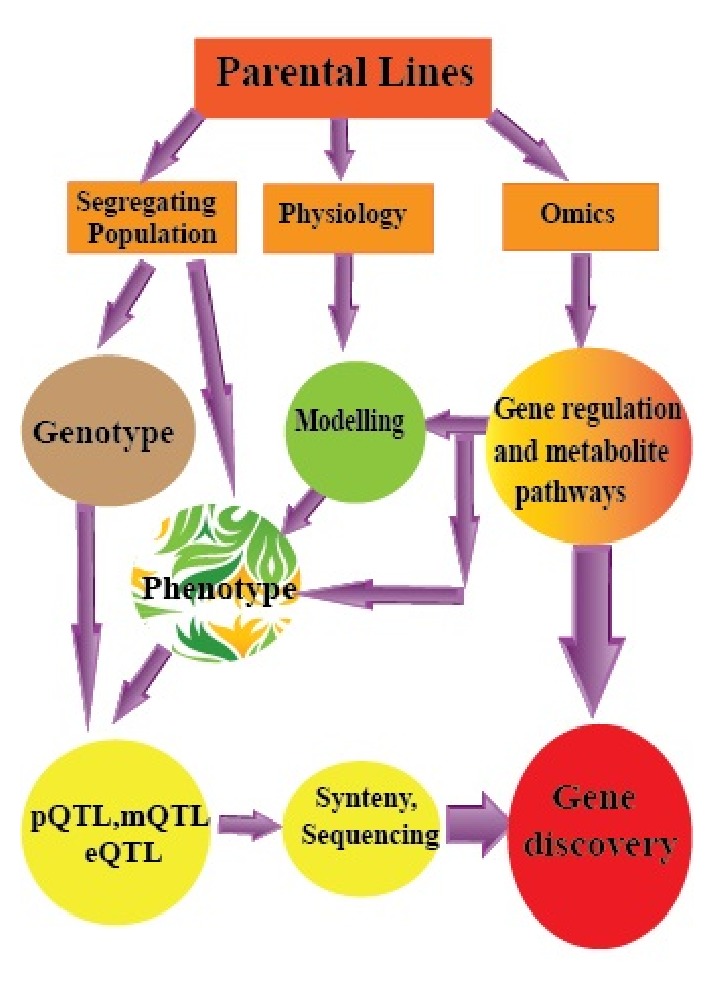
The schematic pathway from selection of parental lines to gene identified. The diagram presents the importance of population development, physiological analysis, phenotyping, and different ‘omics technologies towards discovering a novel responsive gene. Investigation of the drought regime type is the main and first step. Selection suitable germplasm based on target environment, which possibly leads to release majority of loci related to tolerance, is the next issue. The selected lines then will be used to improve segregating populations required for genetic analysis. Selection recombinant lines based on parental omics and physiological traits using different mathematical models offer functional data to choice candidate genes and work on QTLs.

A comprehensive research programme or a multidisciplinary method of integrating the physiology of drought tolerance traits with genomics and genetic tools, including quantitative trait loci, suppression subtractive hybridization, microarrays, and different transgenic crops is needed to enhance drought tolerance in rice. A complete pathway towards improving drought tolerance in rice by discovering candidate genes is illustrated (Figure 2) and briefly described in this paper and includes (i) selecting a specific environment, (ii) creating controlled populations by selecting germplasm adapted to the target environment, (iii) describing the morphological, molecular, and physiological mechanisms involved in the tolerance of the parents, and (iv) integrating this information into models used for QTL analysis and positional cloning (Figure 2).

### 4.3. Focus on a Specific Environment

Drought tolerance experiments should be carried out under a realistic environment and field conditions. Most reports by molecular biologists consider the effects of genes responsible for drought tolerance under unrealistic conditions; they seldom prove their value for breeders or their expected phenotypes under field conditions [145].

On the other hand, different plants have developed their morphology and physiological mechanisms exclusively to tolerate different types of drought stresses. There are different root systems developed by different plants under drought conditions to improve the timing of water absorption from the soil. A deep, compact root system increases yield* via* stored soil moisture by presenting a uniform branching pattern that enhances water use efficiency by reducing the use of water early in the season and increasing access to water during grain filling [146]. Plants from the Mediterranean, which experiences seasonal rainfall along with terminal drought, have large and narrow root systems to extract water from the top layers of soil early in the season [146]. A QTL under drought may have different additive positive, null, or negative effects because of interactions between genotype and the environment [147]. Identified QTLs responsible for drought stress may not be stable in different environmental conditions. They can be classified as constitutive or adaptive based on the stability of their effects across different environmental conditions. Constitutive QTLs are regularly identified across different environments, whereas adaptive QTLs are identified only under particular environmental conditions, and their expression is affected by changing in the environmental factor [148]. Therefore, the occurrence and magnitude of adaptive QTLs differ between experiments, but stable QTLs easily help to understand the same trait under a range of environmental conditions.

Adaptive QTLs are sensitive to any changes in environmental conditions; the QTL gene is regulated by an environmental signal. Alternatively, different responses to environmental changes may be due to the genotype; for example, larger root systems are less affected by nutrient deficit and water shortage than are small root systems. Hence, genes involved in the development of root systems may support QTLs involved in stomatal conductance, abscisic acid content, and biomass accumulation. Moreover, QTLs responsible for flowering time usually affect plant yield under water and nutrient shortages because the period of the plant life cycle influences the intensity and timing of the stresses [149]. Accordingly, the unpredictable effects revealed by most QTLs on yield in diverse environments are not surprising. Thus, describing a target drought scenario is the most important issue for a research programme on drought tolerance.

## 5. Drought Stress Omics

A dataset including alterations in gene expression, protein profiling, and metabolites found in plants under drought conditions should be produced using genomics tools. Monocotyledonous and dicotyledonous plants use the same transcription factors under abiotic stress [150]. The molecular mechanisms in drought-tolerant plants include (i) a signal transduction cascade followed by transcriptional activation and regulation; (ii) protein protection with help of many proteins, such as late embryogenesis abundant-like dehydrins or chaperones such as heat shock proteins; (iii) the accumulation of osmolytes, including mannitol, betaine, proline, trehalose, myo-inositol, and glycine; (iv) the induction of chemical antioxidants, such as glutathione and ascorbic acid; and (v) the reduction of reactive oxygen species (ROS) toxicity by superoxide dismutase and glutathione S-transferase. These different mechanisms may involve several homologous genes that can be identified via transcriptomic experiments on rice lines grown under normal or water stress conditions. Several regulatory mechanisms for drought tolerance are found in many species, but the molecular response to water stress has been experimentally shown [151] to be less consistent because of differences in developmental stage, stress dynamics, and tissue analysed.

### 5.1. The Zinc Finger Protein Family in Rice

In rice, different biological processes are controlled by different transcription factors encoded by* WRKY* genes. Zinc finger proteins, particularly those that regulate stress responses, are widely distributed in plants. The* WRKY *genes are broadly distributed among plants and are present in monocotyledons and dicotyledons. Many WRKY genes play positive or negative regulatory roles in plant responses to different biotic and abiotic stresses [152]. As one of the largest transcription factor families, WRKY transcription factors play key roles in regulating many plant processes, including the response to drought stress [153]. The WRKY proteins contain one or two WRKY domains, which include a WRKY motif and a Zn finger motif. The structure of the DNA-binding domain is the basis for the differentiation of the WRKY family members. The main sequences of WRKY motifs are formed based on variations of the WRKYGQK motif, such as WRKYGKK, WRKYGEK, WKKYGQK, WRKYGRK, WSKYEQK, and WKRYGQK [154]. Two types of C2H2 motifs, (C–X4–5–C–X22–23–H–X1–H) and (C–X5–7–C–X23–H–X1–C), are the Zn fingers found in WRKY proteins [155, 156]. The WRKY proteins are divided into three different groups. Group I contains two WRKY domains and is divided into subgroup Ia, which contains C_2_H_2_ Zn ingers, and subgroup Ib, which contains C_2_HC Zn fingers; group II contains one WRKY domain that is based on short conserved motifs, and this group is further subdivided into five subgroups, a–e, according to their phylogenetic relationship [156]. Group III members have a WRKY domain with a different Zn finger, C_2_HC [155].

**Figure 3:**
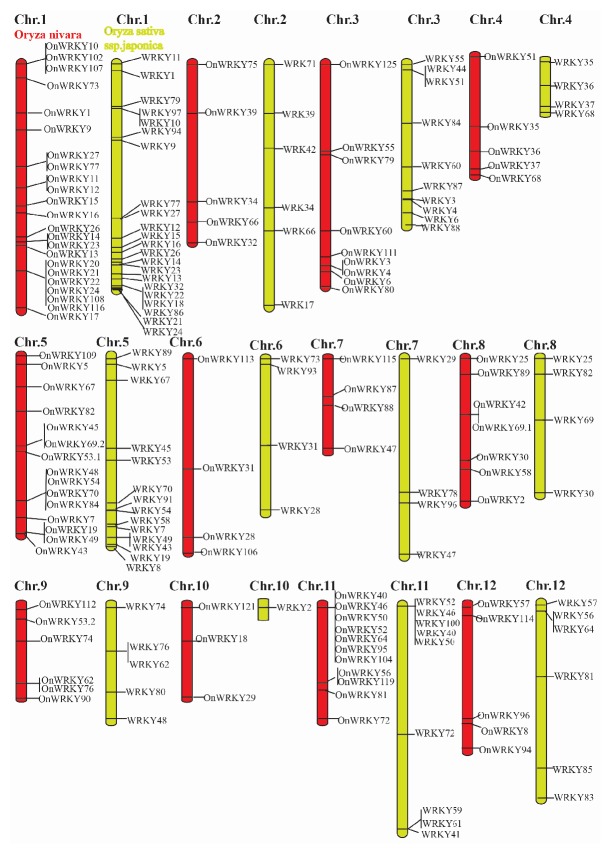
Distribution of the* WRKY *gene family on the* Oryza sativa *ssp.* japonica *chromosomes (right) and wild type* O. nivara* chromosomes chromosome (left). The columns symbolize chromosomes with the name of genes shown on the right.

Rice contains more WRKY protein family members than does* Arabidopsis*. The sequencing and curation of two highly productive rice genomes,* japonica *cv. Nipponbare [157] and wild type* O. nivara*, provide an opportunity to evaluate a large family of transcription factors.* Japonica *cv. Nipponbare presents a wide range of tolerance against environmental stresses [158]. In addition,* O. nivara* displays rich genetic diversity [159] and elite drought tolerance [158]; hence, any differences in the WRKY family that are associated with the regulation of responses toward abiotic stresses, particularly drought stress, would be of interest. The HMMER program [160] was used to identify WRKY genes in* japonica *cv. Nipponbare and* indica *cv. Nivara and to construct a hidden Markov model (HMM) using* Oryza sativa* WKRY proteins [155, 161]. Non-WRKY proteins were removed by screening the results manually for false positives at an e-value>10. A batch BLASTp [162] was performed to identify orthologues by comparing WKRY sequences in* O. Nipponbare* and* O. Nivara*. Mapping these transcription factors to individual chromosomes eased the removal of redundancies and facilitated the identification of 89* WRKY *genes in* japonica *and 97 in wild type* O. nivara* (Figure 3). Two copies of* WRKY46* were identified on chromosomes 11 and 12.* OnWKRY39* and On*WRKY66* seem to be duplicated in* O. Nivara* and have tandem repeats on chromosome 2.* OnWKRY25* appears to be duplicated on chromosome 8 of* O. Nivara. *Similarly, duplications of* OnWKRY40*,* OnWRKY46*,* OnWRK*Y*50*, and* OnWRKY52* could be found in* O. Nivara* on chromosome 11. Lastly,* OnWKRY57* appears to be duplicated in* O. Nivara* on chromosome 12. The inequalities in the WRKY genes and the different loci of some WRKY genes between* Japonica* and* O. Nivara* may contribute to the differences in their ability to cope with different stresses. On the other hand, it may be due to incorrectly identified exons or errors in the detection of intron-exon boundaries or scaffold assembly. Hence, the complete annotation of the WKRY gene family requires additional substantial cDNA cloning and sequencing.

## 6. The Role of Photosynthesis and Photosynthetic Pigments in the Drought Response

**Figure 4:**
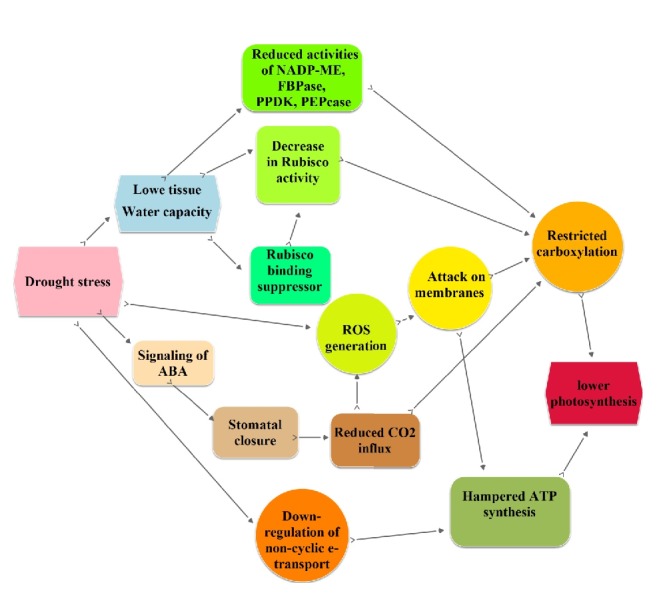
Possible mechanisms of photosynthesis under drought stress.

Among various metabolic processes, photosynthesis is a vital complex process during drought stress. The most important factors that limit photosynthesis are the CO_2_ diffusional restriction because of decreased reductions in photosynthesis, early stomatal closure, and the decreased efficiency of PSII photochemical formation and the biochemical components related to triose phosphate formation. Alterations in any of these components change the overall photosynthetic rate. Mesophyll conductance (*g*m) and stomatal conductance (*g*s) to CO_2_ often decrease under drought stress [163]. Hence, maintenance of the* g*m values under drought stress reflects the tolerance of rice to water deficits [164]. The PSII activity is crucial for providing ATP and power. If PSII activity increases, an excessive decrease in the electron transport chain in the photosynthetic apparatus may occur, thereby stimulating the production of ROS. Accordingly, a balance between demand for photoassimilates and photochemical activity is necessary. Drought stress significantly impairs the activity of PSII in rice flag leaves [165]. This phenomenon occurs because drought induces the degradation of the D1 polypeptide, causing the inactivation of the PSII reaction centre. Drought stress restricts photosynthesis due to a tendency for the activity of Rubisco, a Calvin cycle enzyme, to decrease [166]. Nevertheless, the amount of Rubisco activase promotes ATP-dependent conformational changes, rescues Rubisco sites from dead-end inhibition, and can increase as a protective mechanism under drought stress. Currently, enzymes involved in C_4_ crop photosynthesis have been introduced into rice to alter photosynthesis and plant productivity in response to stress. Transgenic rice plants overexpressing C_4_ photosynthesis enzymes, such as phosphoenolpyruvate carboxylase and pyruvate orthophosphate dikinase, are highly drought tolerant [167] (Figure 4).

Drought leads to various alterations in metabolic functions, one of the most important of which is the reduction or loss of photosynthetic pigment synthesis. This causes reduced light harvesting and reducing power, which act as an energy source for the dark reactions of photosynthesis. Changes in the amounts of photosynthetic pigments are closely associated with biomass and yield [126]. The photosynthetic pigment chlorophyll absorbs light energy and transfers the energy to the photosystem reaction centre. Among various chlorophylls, chlorophylls a and b, which are typically found in higher plants, are susceptible to soil drying. Additionally, other pigments, carotenoids, have various functions in light harvesting, chloroplast photosystem structure, and photoprotection, and can partially promote crop resistance to drought. Decreases in chlorophyll content and the maximum quantum yield of PSII (*F*v/*F*m) have been observed in several studies on rice under drought stress [133, 168]. Decreases in* F*v/*F*m and chlorophyll content have been observed less frequently in autotetraploid lines than in their corresponding diploid lines under drought stress, suggesting that autotetraploid rice is more tolerant of drought stress [169]. The reduction in chlorophyll content may occur because stress impairs pigment degradation or pigment biosynthetic networks and increases lipid peroxidation and the loss of the chloroplast membrane.

## 7. The Biochemical Response of Rice under Drought 

Reduced osmotic potential in the cytosol is as a result of the accumulation of inorganic and organic solutes, which leads to the maintenance of turgor pressure under drought stress [170]. This biochemical procedure is a type of osmotic adaptation that strongly depends on the water stress level. Osmotic adaptation occurs* via* the accumulation of glycine betaine, sucrose, proline, and other solutes in the cytoplasm, promoting water uptake by drying the soil. Proline, an amino acid, is the most widely investigated due to its considerable stress-reducing or function under adverse conditions. Water deficit also induces soluble sugar accumulation [132, 133, 171].

### 7.1. The Role of Proline under Drought

As mentioned above, proline acts as an osmolyte in plants under various adverse conditions [172]. The initial report regarding the role of proline role was made in 1954, when Kemble and MacPherson studied free proline accumulation in rye grasses exposed to stress [173]. The differences in proline accumulation under normal and stress conditions have been reported in rice [128, 133, 136, 174]. Additionally, proline exhibits three main roles under stress, i.e., as a signalling molecule, an antioxidative defence molecule, and a metal chelator [175]. Under drought stress, the accumulation of this amino acid might repair damage by increasing the rate of antioxidant activity [176]. Thus, the proline content can be used as a marker to screen for drought screening tolerance in rice.

### 7.2. The Role of Antioxidants under Drought

An imbalance between the quenching and generation of ROS is the most common phenomenon under drought stress [177]. The ROS include hydroxyl free radicals, singlet oxygen, hydrogen peroxide, and the superoxide radical, and they denature proteins, cause lipid peroxidation, mutate DNA, disrupt cellular homeostasis, and cause cellular oxidative damage. A complex antioxidant system containing enzymatic antioxidants and nonenzymatic molecules protects plants against the adverse effect of ROS. Glutathione (GSH) and ascorbate (AsA) serve as nonenzymatic antioxidants within the cell. Enzymatic antioxidants include catalase (CAT), glutathione reductase (GR), ascorbate peroxidase (APX), superoxide dismutase (SOD), guaiacol peroxidase (GPX), ascorbate-glutathione cycle enzyme, monodehydroascorbate reductase (MDHAR), and dehydroascorbate reductase (DHAR) [178]. These antioxidants are vital ROS-scavenging components in crops, and their expression increases drought tolerance in rice [179]. Increasing levels of drought stress in rice lead to increases in the activity of APX, GSH, AsA [180], GR, MDHAR, SOD, DHAR [181], CAT, and phenylalanine ammonia lyase [171]. The trend for these antioxidant defence enzymes to increase their activity demonstrates their protective activity to counteract the oxidative injury induced by drought stress in rice. The activities of CAT, POD, and SOD can effectively decrease ROS, which ultimately diminishes the negative impact of drought on rice [169, 174].

### 7.3. The Role of Polyamines under Drought

Polyamines (PAs) are small positively charged molecules [182] that are a part of the drought tolerance response in plants [183]. The PAs in plants include spermine (Spm), spermidine (Spd), and putrescine (Put). Polyamines can interact with different signalling networks. Additionally, they regulate ionic homeostasis and osmotic potential and stabilize membranes. The increased PA content of plants exposed to drought is directly correlated with decreased water loss, increased photosynthetic capacity, and improved osmotic detoxification and adjustment. However, the full network of action is poorly understood. The roles of PAs encompass regulating gene expression via maintaining ion balance, facilitating the DNA binding of transcription factors, scavenging radicals, stabilizing membranes, and preventing senescence* via *the conformational transition of DNA and protein phosphorylation [184]. A recent study has demonstrated that rice can promote PA biosynthesis, especially the free forms of Spm and Spd, and conjugate them into insoluble forms in leaves previously exposed to drought stress [185]. Exogenous PAs can reduce drought stress. Their application improves WUE, free proline production, net photosynthesis, leaf water status, soluble phenolics, and anthocyanins and decreases oxidative damage to cellular membranes [138].

## 8. Conclusions

Traditional breeding approaches to select for improved genotypes depend on phenotypic traits, but they are only partially successful because direct selection is hampered by low heritability, genotype-environment interactions, genetic interactions such as epistasis, and polygenic effects. Our knowledge of the drought tolerance mechanism has increased* via *research programmes focused on particular physiological, molecular, and genetic aspects of the drought response. None of these approaches have successfully suppressed the drought susceptibility of rice. However, the integrative plan to link physiology, quantitative genetics, and omics, as recommended above, has been followed by a few research programmes. Accurate phenotyping is critical to screen for superior core mapping populations/collections to identify the most relevant QTLs and isolate candidate gene(s) used in plant breeding. Compared with phenotypic selection, GS selection can reduce the selection time for most traits and has emerged as an important method to predict genotype performance. The* WRKY *genes play important roles in plant development by responding to different biotic and abiotic stresses, and these transcription factors have undergone numerous duplications and deletions in the recent evolution of* Oryza sativa*. Annotation of the* WRKY* family in rice will help determine whether these events are associated with the variation among recent rice cultivars and subspecies or are neutral occurrences in a highly redundant transcription factor family. Combinations of different phenotyping methods and modelling present great potential to swiftly assess the value of definite traits on plant performance. Using models to understand relationships between genotype and phenotype in plants offers an effective platform to create new interactions between genomics/genetics and plant physiology [186]. To overcome the challenge of increasing crop production, systems biology and functional genomics at the crop level should be integrated, and crop physiology will play an important role in achieving this goal. The abundant genomics and genetic analyses of rice offer the opportunity to generate large populations and greatly improved field phenotyping abilities. Nevertheless, genetic studies have not permitted the real dissection of plant responses to drought stress and have not focused on definite drought conditions or regimes. New advances in marker development, sequencing, and genomic analysis have provided the opportunity to reconsider the method of creating populations suitable for analysis and to challenge the precise players in drought tolerance. The rate-limiting and expensive phenotyping step has become a great challenge in analysing drought tolerance and other crop traits. Therefore, the development of rapid and cheap measures to characterize drought response components will effectively improve genetic resolution.

## Figures and Tables

**Table 1:** The QTLs identified for drought tolerance-related traits in rice.

Traits	Number of QTLs	Chromosome/linkage group	Explained Phenotypic variation (%)	Reference
Osmotic adjustment	1 (*OA*_*70*_)	8	Major	[21]
Drought avoidance	17	All except 9	4.4–25.6	[22]
Root traits	18	All chromosomes	1.2–18.5	[23]
Root and related traits	42	All chromosomes	6.0–24.4	[24]
Water stress indicators, phenology and production traits	47	All except 5	5.0–59.0	[25]
Grain yield and other agronomic traits	77	All except 12	7.5–55.7	[26]
Basal root thickness and 100-grain weight	2	4, 6	20.6–33.4	[27]
Coleoptile length and drought resistance index	15	All except 3, 8, 11	4.9–22.7	[28]
Relative growth rate and specific water use	7	2, 4, 5, 6, 7, 8	10.0–22.0	[29]
Grain yield	1(*qtl12.1*)	12	51.0	[30, 31]
Grain yield	2	2, 3	13.0–31.0	[32]
Grain yield	1(*qDTY12.1*)	12	23.8	[33]
Grain yield	1(*qDTY3.2*)	3	23.4	[34]
Grain yield	14	All except 9, 11	13.3	[35]
Filled grain number per panicle	23	All except 12	33.3	[35]
Panicle number per plant	14	All except 1, 3, 8	_	[35]
1000-grain weight	21	All except 12	50.3	[35]
Grain yield (under drought stress)	1(*qDTY2.3*)	2	9.0	[36]
Grain yield (under drought stress)	1(*qDTY2.2*)	2	6.0	[36]
Grain yield	4	3	18.8-31.8	[37]
Physio-morphological	9	4	36.8	[38]
Plant production	24	1,6	14-20.9	[38]
Grain yield	7	1, 2, 3, 9, 12	31-77	[39]

**Table 2:** Candidate genes related to drought stress of model plants and some of major crop species.

Gene	Accession number	Plant species	Function/Plant trait	Reference
AAO3	AT2G27150	*Arabidopsis thaliana*	Stomatal Movement, Ion and Osmotic Homeostasis Regulatory Proteins, ABA Biosynthesis, Hormone Signaling	[40]
ABCG22	AT5G06530	*Arabidopsis thaliana*	Channels and Transporters	[41]
ABF3	AT4G34000	*Arabidopsis thaliana*	Regulatory Proteins, Transcription Factors, bZIP	[42]
ABF4	AT3G19290	*Arabidopsis thaliana*	Regulatory Proteins, Transcription Factors, bZIP	[42]
ABCG40	AT1G15520	*Arabidopsis thaliana*	Stomatal Movement, Ion and Osmotic Homeostasis, Channels and Transporters, ABA Importer	[43]
ABCG25	AT1G71960	*Arabidopsis thaliana*	Stomatal Movement, Ion and Osmotic Homeostasis, Channels and Transporters, ABA Exporter	[44]
ABH1/CBP80	AT2G13540	*Arabidopsis thaliana*	Stomatal Movement, Ion and Osmotic Homeostasis	[45]
ABO1/ELO1	AT5G13680	*Arabidopsis thaliana*	Stomatal Movement, Ion and Osmotic Homeostasis	[46]
ADAP	AT1G16060	*Arabidopsis thaliana*	Regulatory Proteins, Transcription Factors, AP2-Domain	[47]
AGO1	AT1G48410	*Arabidopsis thaliana*	Regulatory Proteins, miRNA	[48]
AHK1	AT2G17820	*Arabidopsis thaliana*	Regulatory Proteins, Signal Transduction, Protein Kinases	[49]
AIRP1	AT4G23450	*Arabidopsis thaliana*	Regulatory Proteins, Post-translational Modification, Ubiquitin Ligases	[50]
DREB2A	AT5G05410	*Arabidopsis thaliana*	Regulatory Proteins, Transcription Factors, DREB	[51]
HYL1	AT1G09700	*Arabidopsis thaliana*	Stomatal Movement, Ion and Osmotic Homeostasis	[52]
LOS5	AT1G16540	*Arabidopsis thaliana*	Stomatal Movement, Ion and Osmotic Homeostasis	[53]
AVP1	AT1G15690	*Arabidopsis thaliana*	Regulatory Proteins, Acid Anhydride Hydrolases	[54]
NCED	AT3G14440	*Arabidopsis thaliana*	Stomatal Movement, Ion and Osmotic Homeostasis, Hormone Signaling, ABA Biosynthesis	[55]
GolS1	AT2G47180	*Arabidopsis thaliana*	Detoxification, Osmolyte Production, Enzymes for Osmolyte Biosynthesis	[56]
GolS2	AT1G56600	*Arabidopsis thaliana*	Detoxification, Osmolyte Production, Enzymes for Osmolyte Biosynthesis	[56]
AnnAt1	AT1G35720	*Arabidopsis thaliana*	Growth Control	[57]
APX2	AT1G07890	*Arabidopsis thaliana*	Detoxification, Removal of ROS, Detoxification Signaling	[58]
AREB1	AT1G45249	*Arabidopsis thaliana*	Regulatory Proteins, Transcription Factors, bZIP	[59]
AtATL78	AT1G49230	*Arabidopsis thaliana*	Post-translational Modification, Ubiquitin Ligases	[60]
AtBG1	AT1G52400	*Arabidopsis thaliana*	Stomatal Movement, Ion and Osmotic Homeostasis, Regulatory Proteins, Hormone and ABA Signaling	[61]
ATHB6	AT2G22430	*Arabidopsis thaliana*	Stomatal Movement, Ion and Osmotic Homeostasis, Regulatory Proteins, Transcription Factors	[62]
AtNF-YB1	AT2G38880	*Arabidopsis thaliana*	Regulatory Proteins, Transcription Factors, NF-Y	[62]
AtrbohD	AT5G47910	*Arabidopsis thaliana*	Stomatal Movement, Ion and Osmotic Homeostasis	[63]
AtrbohF	AT1G64060	*Arabidopsis thaliana*	Stomatal Movement, Ion and Osmotic Homeostasis	[63]
OSM1/SYP61	AT1G28490	*Arabidopsis thaliana*	Stomatal Movement, Ion and Osmotic Homeostasis	[64]
OST1/SRK2E	AT4G33950	*Arabidopsis thaliana*	Stomatal Movement, Ion and Osmotic Homeostasis, Regulatory Proteins, Signal Transduction, Protein Kinases	[63]
CBF4	AT5G51990	*Arabidopsis thaliana*	Regulatory Proteins, Transcription Factors, DREB	[65]
MYC2	AT1G32640	*Arabidopsis thaliana*	Regulatory Proteins, Transcription Factors, MYC	[66]
GORK	AT5G37500	*Arabidopsis thaliana*	Ion and Osmotic Homeostasis, Transporters, Ion Channels, Cation Channel	[67]
GCR1	AT1G48270	*Arabidopsis thaliana*	Stomatal Movement, Ion and Osmotic Homeostasis	[68]
DREB1A/CBF3	AT4G25480	*Arabidopsis thaliana*	Regulatory Proteins, Transcription Factors, DREB	[69]
CBP20	AT5G44200	*Arabidopsis thaliana*	Stomatal Movement, Ion and Osmotic Homeostasis, Regulatory Proteins, miRNA	[70]
CLCc	AT5G49890	*Arabidopsis thaliana*	Ion and Osmotic Homeostasis, Channels and Transporters, Ion and Anion Channel	[71]
CML9	AT3G51920	*Arabidopsis thaliana*	Regulatory Proteins, Signal Transduction	[71]
CPK21	AT4G04720	*Arabidopsis thaliana*	Regulatory Proteins, Signal Transduction, Protein Kinases	[72]
CPK23	AT4G04740	*Arabidopsis thaliana*	Regulatory Proteins, Signal Transduction, Protein Kinases	[73]
CYP707A1	AT4G19230	*Arabidopsis thaliana*	Stomatal Movement, Ion and Osmotic Homeostasis, Regulatory Proteins, Hormone Signaling, ABA Degradation	[74]
CYP707A3	AT5G45340	*Arabidopsis thaliana*	Stomatal Movement, Ion and Osmotic Homeostasis, Regulatory Proteins, Hormone Signaling	[74]
ERD1	AT5G51070	*Arabidopsis thaliana*	Detoxification, Proteases	[72]
GPX3	AT2G43350	*Arabidopsis thaliana*	Detoxification, Removal of ROS, Detoxification Signaling	[73]
HAB1	AT1G72770	*Arabidopsis thaliana*	Ion and Osmotic Homeostasis, Regulatory Proteins, Signal Transduction, Protein Phosphatases	[74]
HD2C	AT5G03740	*Arabidopsis thaliana*	Regulatory Proteins, Histone Modification	[75]
MYB2	AT2G47190	*Arabidopsis thaliana*	Regulatory Proteins, Transcription Factors, MYB	[66]
MRP4	AT2G47800	*Arabidopsis thaliana*	Stomatal Movement, Ion and Osmotic Homeostasis, Channels and Transporters	[76]
SHINE1 (SHN1/WIN1)	AT1G15360	*Arabidopsis thaliana*	Regulatory Proteins, Transcription Factors, AP2-Domain	[77]
SRK2C	AT1G78290	*Arabidopsis thaliana*	Regulatory Proteins, Signal Transduction, Protein Kinases	[77]
MYB60	AT1G08810	*Arabidopsis thaliana*	Regulatory Proteins, Transcription Factors, MYB	[78]
MYB61	AT1G09540	*Arabidopsis thaliana*	Regulatory Proteins, Transcription Factors, MYB	[79]
RPK1	AT1G69270	*Arabidopsis thaliana*	Regulatory Proteins, Signal Transduction, Protein Kinases	[80]
RGS1	AT3G26090	*Arabidopsis thaliana*	Growth Control, Root/Leaf Development, Signal Transduction	[81]
DOR	AT2G31470	*Arabidopsis thaliana*	Stomatal Movement, Ion and Osmotic Homeostasis	[82]
EDT1/HDG11	AT1G73360	*Arabidopsis thaliana*	Growth Control, Root/Leaf Development, Regulatory Proteins, Transcription Factors	[83]
GPA1	AT2G26300	*Arabidopsis thaliana*	Stomatal Movement, Ion and Osmotic Homeostasis	[84]
GTG1	AT1G64990	*Arabidopsis thaliana*	Stomatal Movement, Ion and Osmotic Homeostasis, Regulatory Proteins, Hormone Signaling	[84]
SAD1	AT5G48870	*Arabidopsis thaliana*	Stomatal Movement, Ion and Osmotic Homeostasis	[85]
HARDY	AT2G36450	*Arabidopsis thaliana*	Regulatory Proteins, Transcription Factors, AP2-Domain	[78]
OST2	AT2G18960	*Arabidopsis thaliana*	Stomatal Movement, Ion and Osmotic Homeostasis, Regulatory Proteins, Acid Anhydride Hydrolases	[78]
PIP1;4	AT4G00430	*Arabidopsis thaliana*	Ion and Osmotic Homeostasis, Channels and Transporters, Water Channels	[81]
PIP2;5	AT3G54820	*Arabidopsis thaliana*	Ion and Osmotic Homeostasis, Channels and Transporters, Water Channels	[81]
KAT2	AT4G18290	*Arabidopsis thaliana*	Ion and Osmotic Homeostasis, Channels and Transporters, Ion Channels, Cation Channel	[82]
MYB44	AT5G67300	*Arabidopsis thaliana*	Regulatory Proteins, Transcription Factors, MYB	[83]
NFYA5	AT1G54160	*Arabidopsis thaliana*	Regulatory Proteins, Transcription Factors, NF-Y	[84]
P5CS1	AT2G39800	*Arabidopsis thaliana*	Detoxification, Osmolyte Production, Enzymes for Osmolyte Biosynthesis	[86]
SLAC1	AT1G12480	*Arabidopsis thaliana*	Ion and Osmotic Homeostasis, Channels and Transporters, Ion Channels, Anion Channel	[87]
GTG2	AT4G27630	*Arabidopsis thaliana*	Stomatal Movement, Ion and Osmotic Homeostasis, Regulatory Proteins, Hormone Signaling	[88]
OCP3	AT5G11270	*Arabidopsis thaliana*	Regulatory Proteins, Transcription Factors	[187]
TPS1	AT1G78580	Arabidopsis thaliana	Detoxification, Osmolyte Production, Functional Proteins, Enzymes for Osmolyte Biosynthesis	[78]
PYL9/RCAR1	AT1G01360	*Arabidopsis thaliana*	Stomatal Movement, Ion and Osmotic Homeostasis, Hormone Signaling	[82]
SQE1	AT1G58440	*Arabidopsis thaliana*	Functional Proteins, Phospholipid Metabolism	[83]
HDA19	AT4G38130	*Arabidopsis thaliana*	Regulatory Proteins, Histone Modification	[84]
HDA6	AT5G63110	*Arabidopsis thaliana*	Regulatory Proteins, Histone Modification	[84]
KAT2	AT2G33150	*Arabidopsis thaliana*	Stomatal Movement, Ion and Osmotic Homeostasis, Phospholipid Metabolism	[86]
SLAH3	AT5G24030	*Arabidopsis thaliana*	Ion and Osmotic Homeostasis, Channels and Transporters, Ion Channels, Anion Channel	[87]
PEPCK	AT4G37870	*Arabidopsis thaliana*	Stomatal Movement, Ion and Osmotic Homeostasis	[88]
MIR168A	AT4G19395	*Arabidopsis thaliana*	Regulatory Proteins, miRNA	[47]
FAR1	AT4G15090	*Arabidopsis thaliana*	Regulatory Proteins, Transcription Factors	[79]
FHY3	AT3G22170	*Arabidopsis thaliana*	Regulatory Proteins, Transcription Factors	[79]
AlSAP	LOC_Os07g07350	*Aeluropus littoralis*	Regulatory Proteins, Transcription Factors, Zinc Fingers	[83]
CMO	AT4G29890	*Atriplex hortensis*	Detoxification, Osmolyte Production, Enzymes for Osmolyte Biosynthesis	[84]
BnPIP1	AT3G53420	*Brassica napus*	Ion and Osmotic Homeostasis; Functional Proteins; Channels and Transporters; Water Channels	[86]
BnPtdIns-PLC2	AT3G08510	*Brassica napus*	Functional Proteins; Phospholipid Metabolism	[87]
CAP2	Solyc05g052410	*Cicer arietinum*	Regulatory Proteins, Transcription Factors, AP2-Domain	[88]
SAMDC	Solyc05g010420	*Datura stramonium*	Detoxification, Osmolyte Production, Enzymes for Osmolyte Biosynthesis	[80]
GbRLK	LOC_Os04g56130	*Gossypium barbadense*	Regulatory Proteins, Signal Transduction, Protein Kinases	[81]
GhMKK1	Solyc12g009020	*Gossypium hirsutum*	Regulatory Proteins; Signal Transduction; Protein Kinases	[82]
GmERF3	Solyc06g063070	*Glycine max*	Regulatory Proteins, Transcription Factors, AP2-Domain	[83]
GsZFP1	Glyma10g44160	*Glycine soja*	Regulatory Proteins, Transcription Factors, Zinc Fingers	[84]
CBF4	MLOC_54227	*Hordeum vulgare*	Regulatory Proteins, Transcription Factors, DREB	[86]
CDPK7	LOC_Os03g03660	*Oryza sativa*	Regulatory Proteins, Signal Transduction, Protein Kinases	[87]
CIPK03	LOC_Os07g48760	*Oryza sativa*	Regulatory Proteins, Signal Transduction, Protein Kinases	[88]
CIPK12	LOC_Os01g55450	*Oryza sativa*	Regulatory Proteins, Signal Transduction, Protein Kinases	[88]
